# A novel *TSC1* variant associated with tuberous sclerosis and sacrococcygeal teratoma

**DOI:** 10.1038/s41439-020-00124-8

**Published:** 2020-11-19

**Authors:** Saba Ahmad, Luis Manon, Gifty Bhat, Jerry Machado, Alice Zalan, Nikolas Mata-Machado, Steven Garzon, Akira Yoshii

**Affiliations:** 1 Department of Pediatrics, The Division of Pediatric Neurology, University of Illinois at Chicago, Chicago, IL USA; 2 Department of Pathology, University of Illinois at Chicago, Chicago, IL USA; 3 Department of Pediatrics, The Division of Genetics, University of Illinois at Chicago, Chicago, IL USA; 4 Prevention Genetics, Marshfield, WI 54449 USA; 5 Department of Anatomy and Cell Biology, University of Illinois at Chicago, Chicago, IL USA

**Keywords:** Paediatric neurological disorders, Genetics research, Genetics of the nervous system

## Abstract

Tuberous sclerosis complex (TSC) is an autosomal dominant disease associated with tumors and malformed tissues in the brain and other vital organs. We report a novel de novo frameshift variant of the *TSC1* gene (c.434dup;p. Ser146Valfs*8) in a child with TSC who initially presented with a sacral teratoma. This previously unreported association between TSC and teratoma has broad implications for the pathophysiology of embryonic tumors and mechanisms underlying cellular differentiation.

Tuberous sclerosis complex (TSC) is an autosomal dominant genetic disorder that is caused by loss-of-function variants in the *TSC1* (hamartin) or *TSC2* (tuberin) gene^1–3^. These gene products form a complex^4,5^ and mediate the mammalian target of rapamycin (mTOR) pathway, thereby regulating cell proliferation, differentiation, and growth^6^. Consequently, a pathogenic variant in either gene is associated with tumor and malformed tissue formation termed hamartia, including cortical tubers, subependymal nodules (SEN) and subependymal giant cell astrocytoma in the brain, facial angiofibromas, cardiac rhabdomyomas, renal angiomyolipomas, and lymphangioleiomyomatosis of the lung^7^. Here, we report a child with TSC who initially presented with a presacral cystic teratoma. Genetic testing showed a novel frameshift variant in the *TSC1* gene (c.434dup).

The patient is a male child who was born vaginally at 36 weeks. Fetal ultrasound analyses were normal. Following an uneventful delivery and postnatal course, he was discharged. At home, he was colicky but fed and defecated normally. His development was normal at the initial presentation. However, his parents noticed swelling on his buttock shortly after birth. At the 1-month visit, the patient had a 4 × 3 cm^2^, nontender, fluctuant mass localized between the central sacral area and the left gluteus. A lumbar spine magnetic resonance imaging (MRI) showed a cystic lesion in the pelvis without connection to the spinal canal (Fig. 1a). Laboratory tests at 1 month showed an undetectable serum human chorionic gonadotropin level and 1145 ng/ml of α-fetoprotein (normal for age). The cystic lesion was excised when the patient was 7 weeks old (Fig. 1b, c). Microscopic examination demonstrated the presence of respiratory-type lining (Fig. 1d), brain tissue (Fig. 1e), and cartilage (Fig. 1f), confirming the final diagnosis of a mature cystic teratoma.

**Fig. 1:**
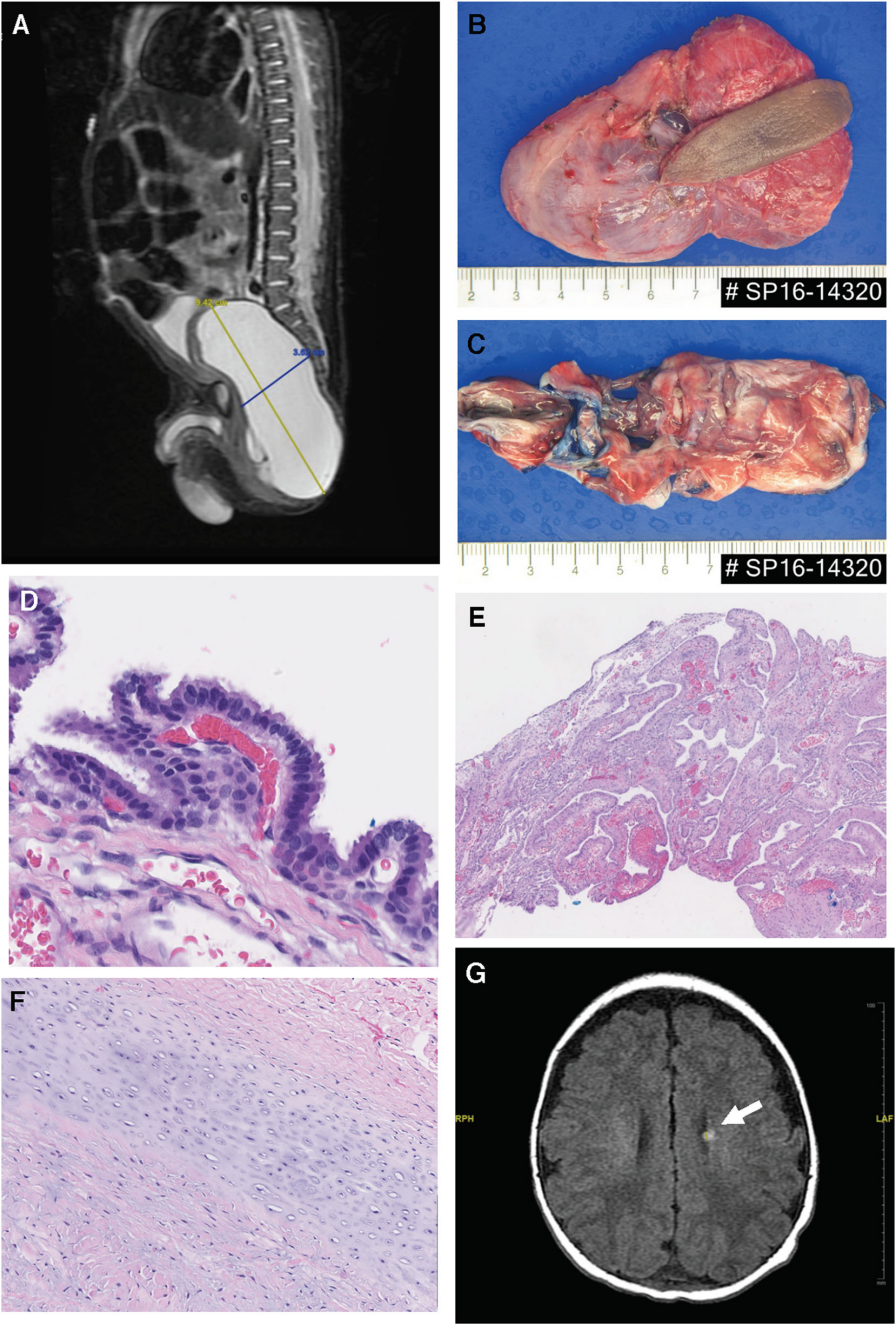
MRI and diagnostic pathological images of the sacrococcygeal teratoma. **a** A lumbar spine MRI showed a cystic lesion in the pelvis without connection to the spinal canal. **b**, **c** The excised tissue was a cystic pink-tan mass (8×6×1cm^3^) weighing ~39g. The lesion contained serous fluid, and its interior surface was smooth. **d**–**f** Microscopic examination demonstrated the presence of respiratory epithelium (**d**), brain tissue (**e**), and cartilage (**f**), confirming the final diagnosis of a mature cystic teratoma. **g** Brain MRI with contrast showed an enhancing SEN (indicated with an arrow) along the left lateral ventricle. The original magnification of histopathology is 40x in (**d**), (**e**), and (**f**).

Presurgical evaluations also included a brain MRI, which showed enhancing SEN along the left lateral ventricle (Fig. 1g). There were also subtle nonenhancing T1 hyperintense nodules within the subcortical white matter. These findings lead to a diagnostic investigation for TSC. On initial skin examination, two hypopigmented macules and numerous “confetti” skin lesions were noted. An ophthalmological examination was unremarkable. Electrocardiogram and echocardiogram were normal, without evidence of arrhythmia or rhabdomyoma. Retroperitoneal ultrasound showed a hyperechoic focus in the inferior pole of the left kidney, but an abdominal MRI was unremarkable.

Next-generation sequencing was applied to the full coding region of the *TSC1* gene using Illumina’s Reversible Dye Terminator platform (Illumina, San Diego, CA USA) and identified a novel frameshift variant (c.434dup), which is predicted to terminate the protein structure prematurely (p.Ser146Valfs*8). This DNA change was confirmed by Sanger sequencing (Fig. 2). The *TSC2* gene was intact. The variant was interpreted as likely de novo because there was no family history of TSC.

**Fig. 2:**
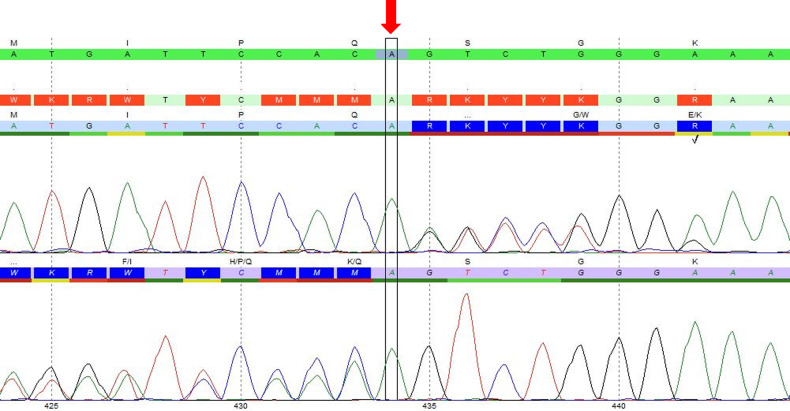
A novel TSC1 gene vatiant. An electropherogram showed a novel variant of the *TSC1* gene (c.434dup, indicated with an arrow).

At 1 year of age, he was able to take independent steps, was interactive, and articulated a few words. On follow-up evaluation, some developmental delays were noted, primarily in speech, and the patient received occupational and speech therapies. At the age of 2, he had brief and frequent staring episodes. A video electroencephalogram revealed right temporal spikes. He was treated with levetiracetam and had no further staring episodes. A follow-up spinal MRI at 2 years and 4 months was negative for tumor regrowth.

Our patient had clinical findings of hypopigmented macules, multiple confetti lesions, and SENs, which prompted molecular testing and confirmed the diagnosis of TSC. He further developed seizures and developmental delay, which are clinical symptoms associated with TSC. Genetic testing revealed a novel heterozygous *TSC1* pathogenic variant: c.434dup. To our knowledge, this variant has not been reported previously. This variant is predicted to result in a frameshift change (p.Ser146Valfs*8) and premature protein termination and is hence considered to be disease causing.

The radiological and pathological findings noted in our patient were consistent with sacrococcygeal mature teratoma. Teratomas are a type of germ cell tumor composed of somatic tissues derived from two or three of the germ cell layers, including the ectoderm, endoderm, and mesoderm. Mature teratoma is a subclassification of teratomas primarily composed of tissues found in a mature adult, as opposed to the fetal tissue that occurs in immature teratomas. Fully differentiated tissues, such as epidermal, central nervous system, muscle, adipose, gut, and respiratory components with little or no mitotic activity, have been identified in mature teratomas. Malignant changes in cystic teratomas have been reported. In the present case, the final pathology report concluded no morphological evidence of an immature teratoma, with no malignant change.

TSC is also rarely associated with chordoma, which is a bone tumor arising from remnants of the embryonic tissue notochord and is present in the sacrococcygeal region as well as the clivus and cervical spine^8–17^. Among these reported cases, two pathogenic variants in *TSC1* (NM_000368.4: c.1825G > T, p.(Glu609*) reported as G2045T in exon 1510; a 9-bp de novo in-frame deletion, NM_000368.4: c.181_189del, p.(Thr62_Leu64del) reported as 402_410delCTGACCACC11 using alternative nomenclature) and one pathogenic variant in *TSC2* (NM_000548.4: c.3028C > T, p.(Gln1010*) in exon 2611) have been documented. In one study, 65% of sporadic chordomas exhibited activation of the mTOR pathway^18^. However, the current case report documents an association between TSC due to a novel pathogenic variant in *TSC1* and sacrococcygeal teratoma. Interestingly, embryonic stem cells (ESCs) derived from *Tsc2*^−^^*/−*^ *Eker* rats differentiated into teratomas when the ESCs were transplanted into nonobese diabetic/severe-combined immunodeficiency mice^19,20^. Therefore, the potential role of the mTOR pathway in the pathophysiology of teratoma may have broader implications for stem cell biology.

## Data Availability

The relevant data from this Data Report are hosted at the Human Genome Variation Database at 10.6084/m9.figshare.hgv.2924.
